# Low HDL-C can be a biomarker to predict persistent severe AKI in septic patients? A retrospective cohort study

**DOI:** 10.1186/s40001-023-01513-9

**Published:** 2023-12-05

**Authors:** Wei Jiang, Lin Song, Weilei Gong, Yaosheng Zhang, Kerang Shi, Ting Liao, Chuanqing Zhang, Jiangquan Yu, Ruiqiang Zheng

**Affiliations:** 1 Medical College, Yangzhou University, Yangzhou, 225001 China; 2 Department of Critical Care Medicine, Clinical Medicine College, Yangzhou University and Intensive Care Unit, Northern Jiangsu People’s Hospital, Yangzhou, 225001 China; 3 School of Pharmaceutical Sciences and Institute of Materia Medica, Shandong First Medical University and Shandong Academy of Medical Sciences, Jinan, 250000 China; 4 School of Clinical and Basic Medicine, Shandong First Medical University and Shandong Academy of Medical Sciences, Jinan, 250000 China

**Keywords:** High-density lipoprotein cholesterol, Lipoproteins, Persistent acute kidney injury, Sepsis

## Abstract

**Purposes:**

Low HDL-C is associated with an increased risk of sepsis-associated AKI and subsequent decline in eGFR. HDL-C possesses anti-inflammatory, antioxidant, and endothelial repair-promoting properties. The use of Apo A-I mimetic peptides, which are the main structural components of HDL-C, has been shown to improve renal function in animal models of sepsis. However, the diagnostic value of low HDL-C in persistent sepsis-associated AKI remains unclear.

**Methods:**

This is a retrospective cohort study based on MIMIC IV (V 2.2). The study population consisted of all adult septic patients admitted to the Beth Israel Deaconess Medical Center Intensive Care Unit from 2008 to 2019, with plasma HDL-C measured within 24 h of ICU admission. The primary endpoint was persistent severe sepsis-associated acute kidney injury (SA-AKI) and the secondary endpoint is kidney replacement therapy (KRT). Logistic regression was used to assess the correlation between HDL-C and persistent severe SA-AKI and KRT, and receiver operating characteristic (ROC) curve analysis was performed to evaluate predictive ability.

**Results:**

A total of 604 cases of SA-AKI patients were included in the analysis, among which 88 cases (14.5%) experienced persistent severe SA-AKI. The median (IQR) HDL-C level in the group with persistent severe SA-AKI was lower (33.0 [24.0–45.5]) compared to the non-persistent severe SA-AKI group (42.0 [31.0–53.0]). However, HDL-C showed poor discriminatory ability with an AUROC [95%CI] of 0.62 [0.56–0.69]. Clinical prediction models based on serum creatinine concentration, 24-h creatinine change, APSIIIscore, lactate levels, APTT, and heart rate performed well in predicting persistent severe SA-AKI with an AUROC [95%CI] of 0.876 [0.84–0.91]. However, adding HDL-C to this model did not improve predictive performance.

**Conclusions:**

The plasma HDL-C measured within 24 h after admission to the ICU does not provide a good prediction for persistent severe SA-AKI, and it does not improve the clinical predictive ability compared to conventional variables.

**Supplementary Information:**

The online version contains supplementary material available at 10.1186/s40001-023-01513-9.

## Introduction

Acute kidney injury (AKI) is a highly prevalent disease worldwide, with sepsis being the most common factor leading to AKI in critically ill patients, accounting for 40% of cases [1]. A recent study estimated that 68% of sepsis patients have AKI upon admission, with severe AKI occurring in 40% of cases and subsequent kidney replacement therapy (KRT) being required during their stay in the intensive care unit (ICU) for 27% of them [2]. The development of sepsis-related AKI is associated with higher mortality rates and longer hospital stays [3].

In clinical practice, the identification of persistent AKI is of great clinical significance. Firstly, the duration of AKI is closely related to patient prognosis and the risk of end-stage renal failure. Recent evidence has shown that two-thirds of AKI patients recover kidney function within 3–7 days, while those with persistent AKI have significantly lower one-year survival rates [4]. Additionally, the persistence of AKI also increases the risk of developing chronic kidney disease (CKD) in individuals [5]. Early identification and active intervention in individuals at risk for persistent AKI can potentially impact its progression to CKD [5, 6]. Secondly, the duration of AKI is closely associated with the need for kidney replacement therapy (KRT). Studies have indicated that some patients may benefit from starting KRT earlier, while others may not require such treatment as they quickly regain kidney function [7]. Therefore, predicting short-term reversibility of AKI may help assess the likelihood of needing KRT and ultimately determine the optimal timing to initiate it. Given the importance of identifying persistent renal injury, new tools including urinary biomarkers and renal Doppler ultrasound have recently been evaluated for this purpose [8, 9].

High-density lipoprotein (HDL) possesses anti-inflammatory, antioxidant, and endothelial repair-promoting properties, participating in the regulation of various pathological processes that influence the progression of sepsis associated acute kidney injury (SA-AKI). HDL increases liver clearance of LPS through scavenger receptor class B type 1 (SR-B1) [10], thereby alleviating LPS-TLR-4-mediated renal tubular injury [11]. HDL may also affect the development of AKI during sepsis by directly and indirectly inhibiting inflammatory responses. HDL can also suppress inflammation during sepsis by inducing the expression of transcription factor 3, reducing the production of IL-6 and TNF-a in macrophages [12–18]. In addition, HDL can protect endothelial function by inhibiting the expression of intercellular adhesion molecule 1(ICAM-1) and stimulating endothelial nitric oxide synthase (eNOS) activity [19, 20]. The use of Apo A-I mimetic peptide, a major component of HDL structure, is associated with improved renal function in septic animal models [21]. In population studies, it has been found that low levels of high-density lipoprotein during sepsis are associated with an increased risk of sepsis-associated AKI and subsequent decrease in estimated glomerular filtration rate(eGFR) [22]. These results indicate that high-density lipoprotein may be a marker of kidney injury during sepsis, but the correlation between low HDL-C and persistent renal dysfunction is still unknown.

The purpose of this study is to determine whether plasma HDL-C measured within 24 h after admission to the ICU can predict persistent severe acute kidney injury and KRT. The secondary objective is to evaluate the potential use of HDL-C in combination with routine clinical data.

## Method

### Data source

This is a retrospective cohort study using the MIMIC-IV (version 2.2) database to investigate different populations. The MIMIC-IV database is a publicly available multi-parameter intensive care unit (ICU) database provided by the Massachusetts Institute of Technology (MIT). It includes critically ill patients admitted to the ICU at Beth Israel Deaconess Medical Center in Boston, Massachusetts, from 2008 to 2019 [23]. Since this study is based on analysis of a third-party anonymous public database and has obtained institutional review board approval in advance, ethical review is not required. To gain access to this database, we have completed the online training course and Protecting Human Research Participants exam offered by the National Institutes of Health (No. 5478440).

### Study population and definitions

This study selected adult patients from the MIMIC-IV database who were admitted to the ICU once and had HDL-C measurements within 24 h after admission. Patients who met the criteria for sepsis 3.0 were included in this study, with inclusion criteria being: presence of infection and Sequential Organ Failure Assessment (SOFA) score ≥ 2 [24]. The diagnosis criteria for AKI followed Kidney Disease: Improving Global Outcomes(KDIGO) standards: an increase in Scr exceeding 26.5 μmol/L (0.3 mg/dl) within 48 h; a rise in serum creatinine by 50% compared to baseline within 7 days; urine output < 0.5 ml/(kg·h) sustained for more than 6 h [25]. This study excluded patients who received KRT treatment immediately (within 6 h) after ICU admission, those with stage CKD-5, kidney transplant recipients, or known infections of human immunodeficiency virus or active hepatitis. The primary endpoint of this study was persistent AKI, defined as developing into stage 3 AKI during ICU stay and lasting for more than 72 h. Patients who died or underwent KRT before reaching the full duration of stage 3 AKI (< 72 h) were also considered to have persistent severe AKI [26].

### Data extraction and preprocessing

Extracted variables from the database using PostgreSQL 14.5 include demographic information, vital signs, medical history, laboratory test results, scoring data, and prognosis data for patients. All comorbidities are diagnosed based on International Classification of Diseases (ICD) codes from the 9th and 10th editions. HDL-C and other laboratory test results are obtained within 24 h after admission to the ICU. Considering that laboratory data is measured multiple times within a 24-h period, this study extracted the worst value for each day. For missing experimental data that accounts for less than 15% of the total population, multiple imputation methods were employed [27].

### Statistical methods

Normally distributed continuous data were presented as mean ± standard deviation (X ± s), while non-normally distributed continuous data were presented as median (interquartile range) [Median (IQR)]. Group comparisons were performed using *t*-tests or rank-sum tests. Categorical data were presented as frequency (*N*) and percentage (%), with group comparisons analyzed using chi-square tests. Variables that were statistically significant on univariate analysis were included in multivariate analysis. Multivariable analysis was performed with a logistic regression model. We considered *p* < 0.05 to indicate statistical significance. The ability to predict persistent severe AKI as well as KRT was assessed using receiver operating characteristic curve (ROC) analysis. All analyses were performed using R software version 4.62.

## Result

### Population characteristics

**Fig. 1:**
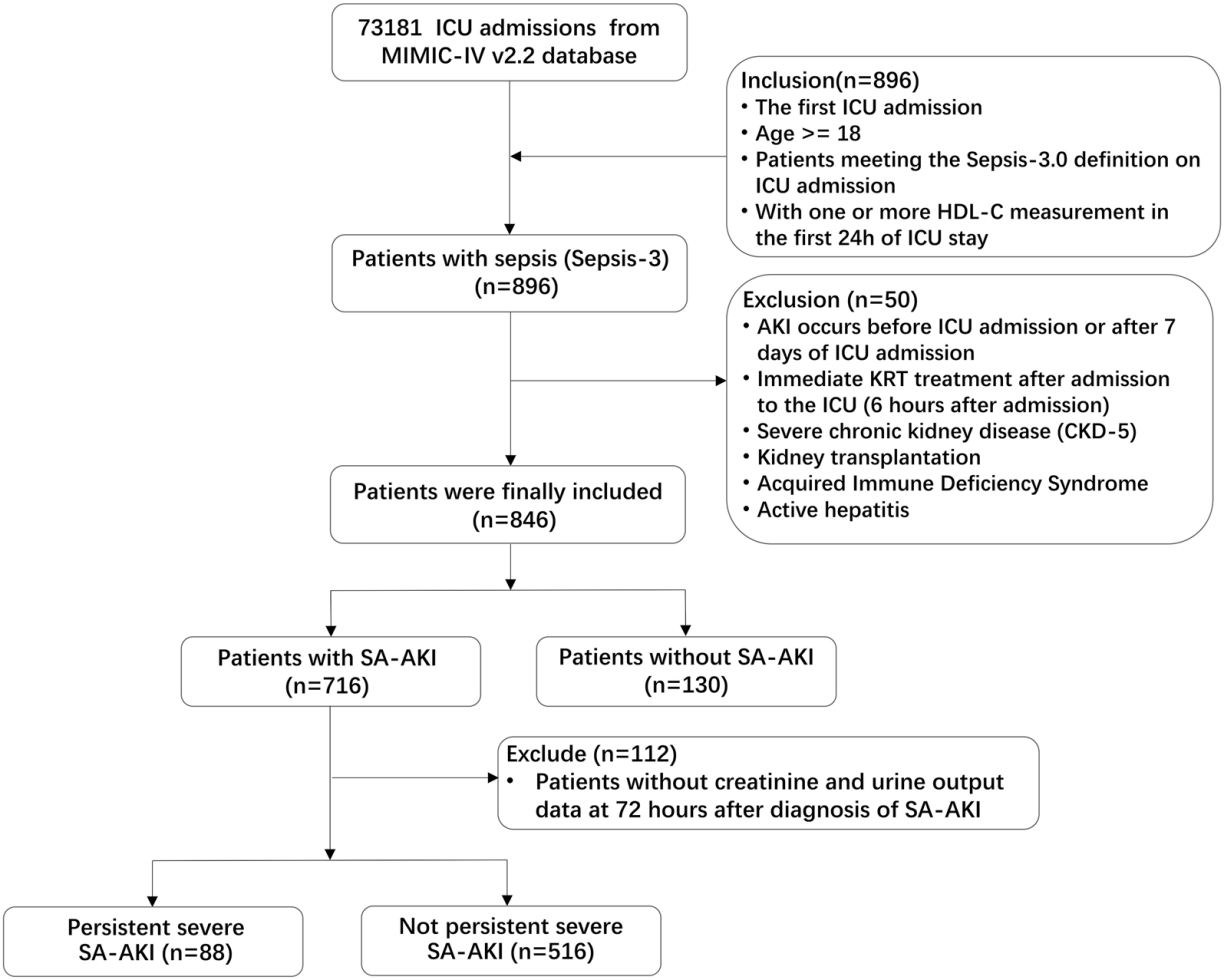
Study flow chart. ICU, intensive care unit; HDL-C, High density lipoprotein cholesterol; CKD, Chronic kidney disease; KRT, kidney replacement therapy; SA-AKI, Sepsis associated Acute kidney injury

This study included 846 patients with sepsis, of which 716 were diagnosed with SA-AKI. After excluding 112 patients who did not have plasma creatinine and urine output measurements within 72 h after SA-AKI diagnosis, a total of 604 SA-AKI patients were finally included for analysis. Among them, 88 cases (14.5%) experienced persistent severe SA-AKI (stage 3), while 516 cases (85.4%) had non-persistent severe SA-AKI (Fig. 1). The main characteristics of the study population are shown in Table 1. The demographic data between the two groups were similar. The prevalence of non-persistent severe SA-AKI was higher in patients with hypertension (51.0% vs. 38.6%, *p* = 0.043) and cerebrovascular disease (50.4% vs. 31.80%, *p* = 0.002). The incidence of coronary atherosclerotic heart disease was higher in patients with persistent severe SA-AKI (58.0% vs. 39.3%, *p* = 0 0.002). Patients with persistent severe SA-AKI had higher scores for disease severity, such as median [IQR] SOFA score on the day of ICU admission (11.0 [7.0–14.0] vs 6.0 [4.0–8.0], *p* < 0.001) and median [IQR] APS III score (81.5 [61.8–97.2] vs 51.00 [39.8–66.0], *p* < 0.001).Patients with persistent severe SA-AKI present with more severe kidney injury, as reflected by higher median [IQR] serum creatinine levels on the day of admission to the ICU (2.40 [1.58–3.73] vs. 1.20 [0.90–1.60] mg/dl, *p* < 0.001) and higher median [IQR] blood urea nitrogen levels (34 [23.8–48.0] vs 22.0[16.30–33.0] mg/dl, *p* < 0.001).

**Table 1:** Baseline characteristics of patients grouped by persistent severe SA-AKI

*N*	Not persistent severe SA-AKI	Persistent severe SA-AKI	*p*. overall
	*N* = *516*	*N* = *88*	
Age (years)	69.5 [59.0;80.5]	67.4 [54.7;80.0]	0.364
Gender, Male, *n*%	311 (60.3%)	57 (64.8%)	0.495
Ethnicity, *n*%			0.184
White	306 (59.3%)	42 (47.7%)	
Asian	5 (0.97%)	1 (1.14%)	
Black	40 (7.75%)	8 (9.09%)	
Other	165 (32.0%)	37 (42.0%)	
Admission type, *n*%			0.600
Emergency	6 (1.16%)	0 (0.00%)	
Non-emergency	510 (98.8%)	88 (100%)	
Charlson_comorbidity_index	6.00 [4.00;8.00]	6.00 [4.00;8.00]	0.771
*Comorbidities, n%*			
Hypertension	263 (51.0%)	34 (38.6%)	0.043
Coronary_atherosclerosis	203 (39.3%)	51 (58.0%)	0.002
Chronic_heart_failure	189 (36.6%)	41 (46.6%)	0.097
Chronic_liver_disease	58 (11.2%)	12 (13.6%)	0.639
Diabetes	159 (30.8%)	32 (36.4%)	0.362
Chronic_kidney_disease	96 (18.6%)	21 (23.9%)	0.314
Peripheral_vascular_disease	51 (9.88%)	8 (9.09%)	0.970
Cerebrovascular_disease	260 (50.4%)	28 (31.8%)	0.002
Chronic_pulmonary_disease	88 (17.1%)	12 (13.6%)	0.521
Cancer	43 (8.33%)	5 (5.68%)	0.524
*Severity scale*			
SOFA Score	6.00 [4.00;8.00]	11.0 [7.00;14.0]	< 0.001
Non renal SOFA Score	5.00[3.00;7.00]	9.00[5.00;11.00]	< 0.001
APSIII Score	51.0 [39.8;66.0]	81.5 [61.8;97.2]	< 0.001
*Vital signs*			
Heart rate (beats/min)	103 [90.0;115]	110 [94.0;128]	0.001
RR (times/min)	27.0 [24.0;31.0]	29.0 [25.0;33.0]	0.109
MAP (mmHg)	62.0 [55.0;69.0]	55.0 [46.0;63.2]	< 0.001
Spo2 (%)	93.0 [91.0;95.0]	93.0 [88.0;95.0]	0.047
Temperature (℃)	36.6 [36.2;36.8]	36.4 [35.8;36.7]	0.025
*Laboratory tests*			
BUN (mg/dL)	22.0 [16.0;33.0]	34.0 [23.8;48.0]	< 0.001
Creatinine (mg/dL)	1.20 [0.90;1.60]	2.40 [1.58;3.73]	< 0.001
Δ_Scr (mg/dL)	0.00 [-0.20;0.10]	0.35 [-0.10;1.02]	< 0.001
Sodium (mEg/L)	141 [138;144]	140 [138;144]	0.951
Potassium (mEq/L)	4.40 [4.10;4.80]	4.90 [4.30;5.80]	< 0.001
Chloride (mEq/L)	106 [103;109]	105 [101;111]	0.454
Aniongap (mmol/L)	17.0 [14.0;19.0]	21.0 [17.8;26.2]	< 0.001
INR	1.20 [1.10;1.50]	1.50 [1.20;2.30]	< 0.001
PT(s)	13.8 [12.5;16.3]	16.3 [13.4;25.0]	< 0.001
APTT(s)	32.8 [27.4;54.9]	48.1 [32.3;92.7]	< 0.001
Platelets (k/uL)	176 [133;228]	148 [96.5;225]	0.015
WBC (k/uL)	13.5 [10.1;17.3]	15.4 [12.0;21.6]	0.002
Hemoglobin (g/uL)	11.1 ± 2.37	11.0 ± 2.44	0.781
RBC (k/uL)	3.73 [3.21;4.22]	3.35 [2.72;4.14]	0.009
RDW	14.2 [13.5;15.4]	14.6 [13.9;16.3]	< 0.001
Bilirubin_total (mg/dl)	0.70 [0.40;1.20]	0.90 [0.58;2.10]	0.002
Albumin (g/dl)	3.50 [2.90;3.90]	3.00 [2.60;3.40]	< 0.001
Ast (U/L)	46.0 [26.0;150]	193 [43.5;785]	< 0.001
Alt (U/L)	30.5 [18.0;94.5]	79.5 [28.0;294]	< 0.001
Alp (U/L)	76.0 [60.0;101]	89.5 [64.8;127]	0.002
Glucose (mg/dL)	158 [125;216]	216 [157;350]	< 0.001
Lactate (mmol/L)	1.60 [1.10;2.70]	2.65 [1.58;4.82]	< 0.001
HDL_C (mg/dl)	42.0 [31.0;53.0]	33.0 [24.0;45.5]	< 0.001
*Interventions, n%*			
KRT	19 (3.68%)	44 (50.0%)	< 0.001
Mechanical ventilation			0.001
None	28 (5.43%)	2 (2.27%)	
Invasive	344 (66.7%)	76 (86.4%)	
Non invasive	144 (27.9%)	10 (11.4%)	
Vasopressor use	208 (40.3%)	66 (75.0%)	< 0.001
Diuretic	342 (66.3%)	55 (62.5%)	0.569
Statin	291 (56.4%)	42 (47.7%)	0.163
Aminoglycoside	40 (7.75%)	32 (36.4%)	< 0.001
ACEI/ARBs	99 (19.2%)	5 (5.68%)	0.003
*Prognosis*			
Length of hospital stay (d), median (IQR)	10.7 [7.13;16.9]	11.8 [6.70;17.8]	0.984
Length of ICU stay (d), median (IQR)	5.35 [3.62;9.70]	6.82 [4.58;11.4]	0.001
Death in hospital, *n*%	44 (14.0%)	59 (39.1%)	< 0.001
Death in ICU, *n*%	29 (9.21%)	48 (31.8%)	< 0.001

*SA-AKI* Sepsis Associated Acute kidney injury, *APS III* Acute Physiology Score III, *SOFA* Sequential Organ Failure Assessment, *KRT* Kidney replacement therapy, *RR* Respiratory rate, *MAP* mean arterial pressure, *ACEI/ARBs* Angiotensin-converting enzyme inhibitors/angiotensin receptor blockers, *APTT* Activated partial thromboplastin time, *PT* Prothrombin time, *BUN* Blood urea nitrogen, *WBC* White blood cell, *RBC* Red blood cell, *ΔScr* Changes in serum creatinine within 24 after ICU admission, *RDW* Red blood cell distribution width, *Ast* Aspartate transaminase, *Alt* Alanine transaminase, *Alp* Alkaline phosphatase, *ICU* Intensive care unit, *HDL-C* High density lipoprotein cholesterol

### Differences between the high HDL-C group and the low HDL-C group

According to the median value of HDL-C in the population, we divided HDL-C into low HDL-C group and high HDL-C group (Table 2). Compared to the high HDL-C group, the low HDL-C group had a higher incidence of persistent severe SA-AKI (18.7% vs. 10.5%, *P* = 0.007), KRT rate (14.30% vs. 6.58%, *P* = 0.003), and usage of vasoactive drugs (50.0% vs. 40.8%, *P* = 0.028). The low HDL-C group also had higher SOFA scores (7.0 [5.0–11.0] vs. 5.5 [4.0–7.0], *P* < 0 0.001), APS III scores (57.0 [42.0–82.0] vs. 52.0[41.0–66.00], *P* = 0.001) and serum creatinine levels (1.30[0.90–2.20] vs.1.20[0.80 -1.70] mg/dl, *P* = 0.04) (Additional file 1: Fig. S1).

**Table 2:** Differences between the high HDL-C group and the low HDL-C group

*N*	Low HDL-C	High HDL-C	*p* overall
	*N* = *300*	*N* = *304*	
Persistent severe SA-AKI, *N*%	56 (18.7%)	32 (10.5%)	0.007
APSIII	57.0 [42.0;82.0]	52.0 [41.0;66.0]	0.001
SOFA Score	7.00 [5.00;11.0]	5.50 [4.00;7.00]	< 0.001
Non renal SOFA Score	6.00 [4.00;9.00]	4.50 [3.00;6.00]	< 0.001
RRT, *N*%	43 (14.3%)	20 (6.58%)	0.003
Vasopressor, *N*%	150 (50.0%)	124 (40.8%)	0.028
Scr (mg/dl)	1.30 [0.90;2.20]	1.20 [0.80;1.70]	0.004

*SA-AKI* Sepsis associated acute kidney injury, *APS III* Acute Physiology Score III, *SOFA* Sequential Organ Failure Assessment, *KRT* Renal replacement therapy, *HDL-C* high density lipoprotein cholesterol, *Scr* Serum creatinine

### Ability of HDL-C to predict persistent severe SA-AKI

**Fig. 2:**
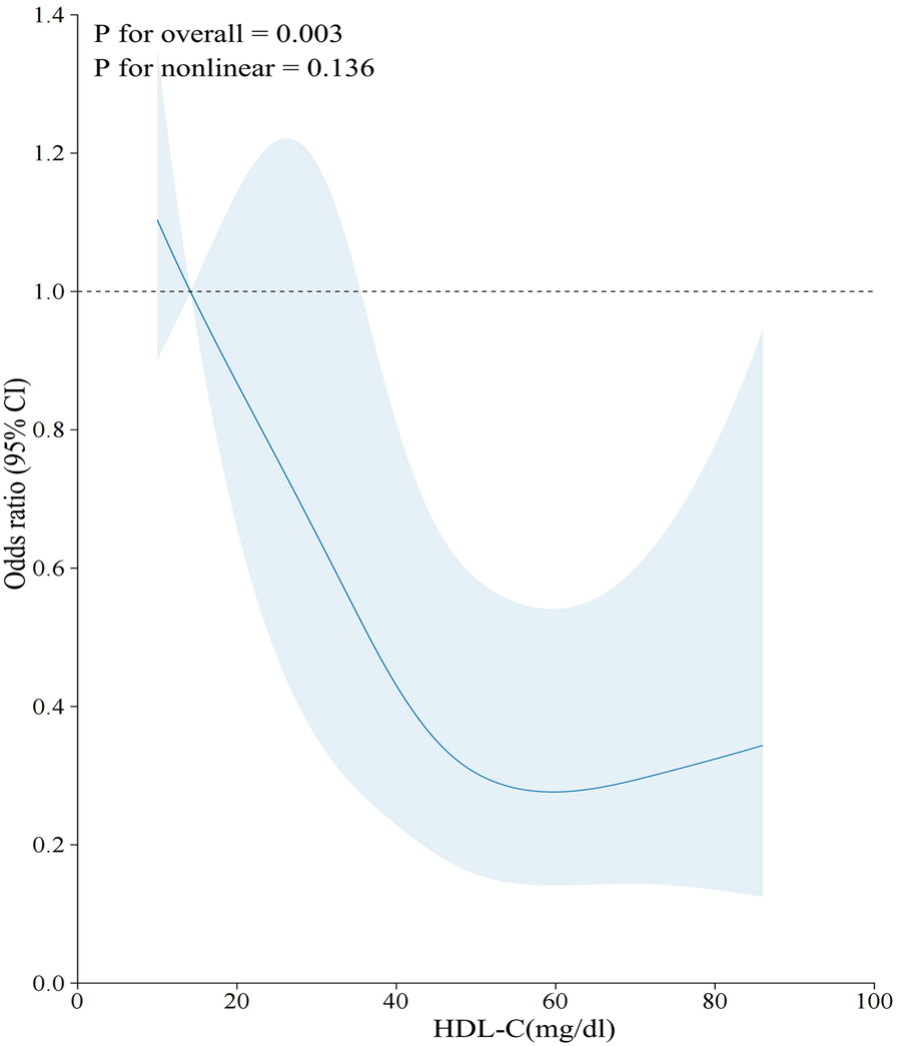
Association between HDL-C and persistent severe SA-AKI using a Restricted Cubic Spline Regression Model. HDL-C, High density lipoprotein cholesterol

**Fig. 3:**
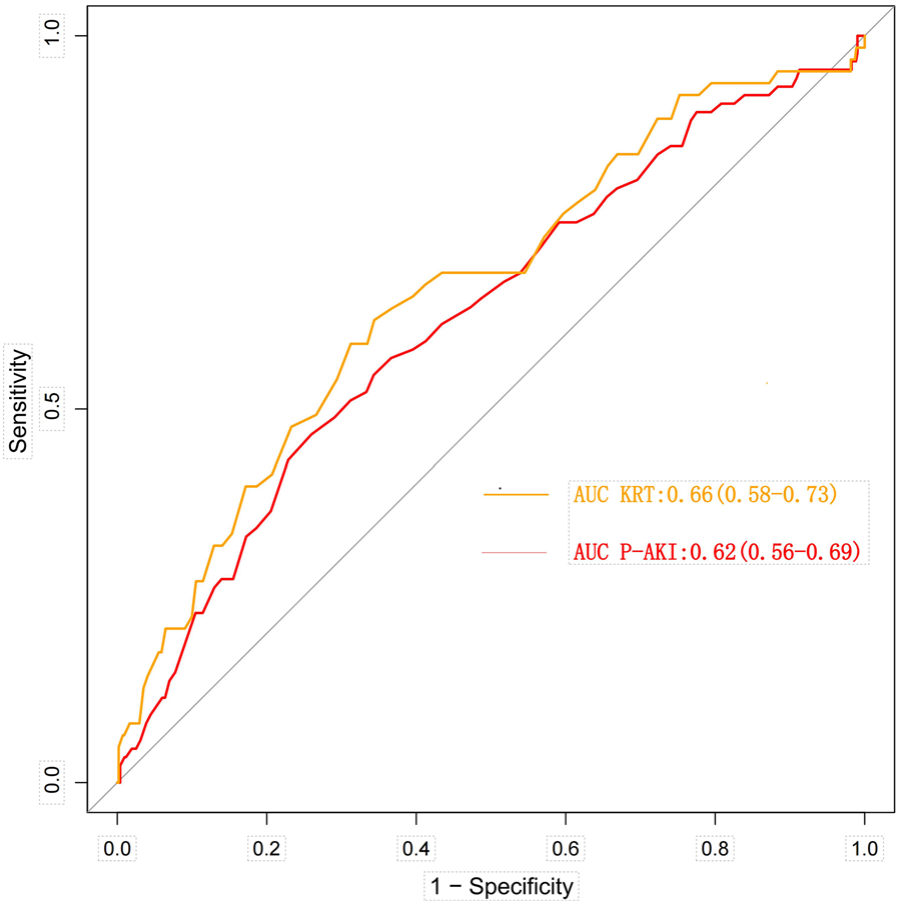
Receiver operating characteristic (ROC) curve of HDL-C to predict persistent SA-AKI and KRT in the overall population. AUC, area under the ROC curve; HDL-C, High density lipoprotein cholesterol; KRT, kidney replacement therapy; P-AKI, Persistent sepsis associated Acute kidney injury

**Fig. 4:**
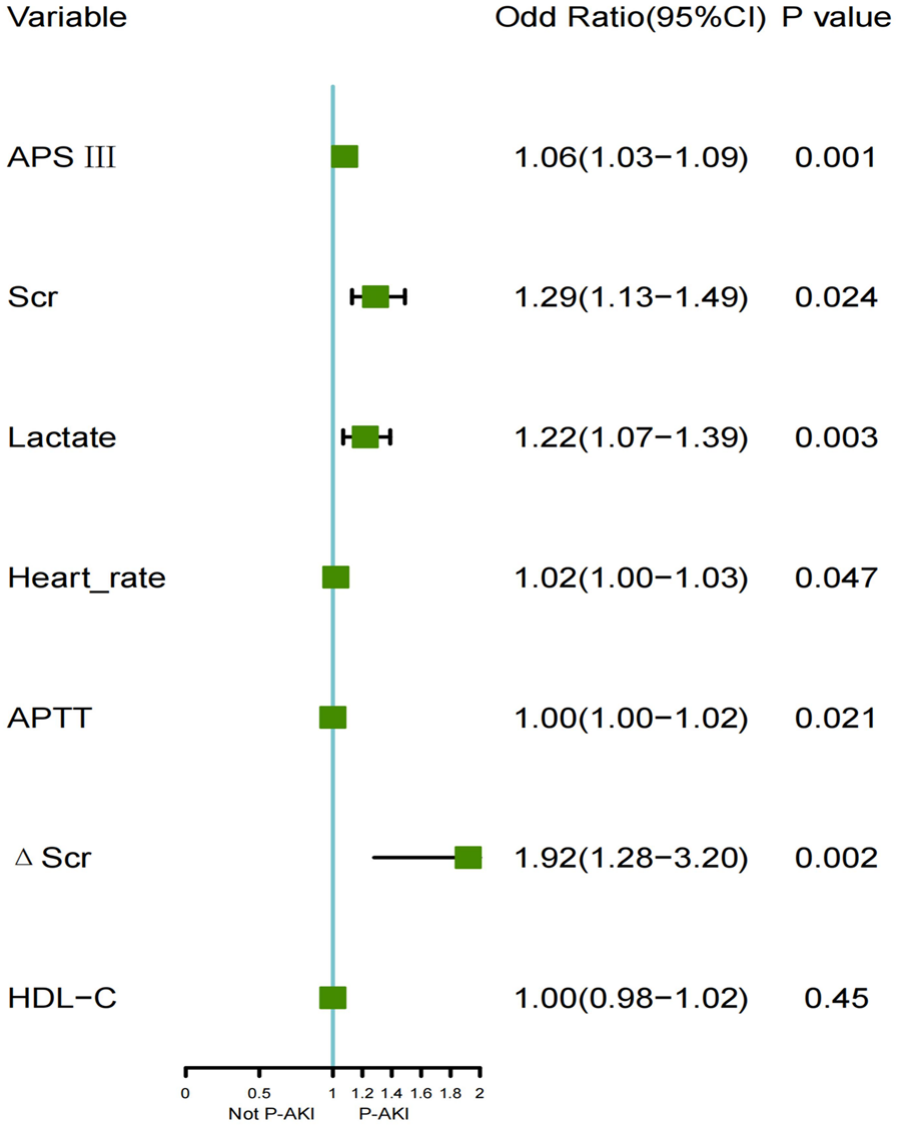
Multivariate analysis of persistent severe SA-AKI. HDL-C, High density lipoprotein cholesterol; APSIII, Acute physiology scoreIII; Scr,Serum creatinine; ΔScr, Changes in serum creatinine within 24 after ICU admission; APTT, Activated Partial Thromboplastin Time; P-AKI, Persistent severe sepisis associated acute kidney injury

In the included SA-AKI population, HDL-C was negatively correlated with serum creatinine (*R* = − 0.12, *P* = 0.0041) and blood urea nitrogen (*R* = − 0.11, *P* = 0.0066) (Additional file 1: Fig. S2A, B). The lower the HDL-C level, the higher the levels of serum creatinine and blood urea nitrogen. The HDL-C in the persistent severe SA-AKI group was significantly lower than that in the non-persistent severe AKI group (Additional file 1: Fig. S2C). RSC analysis showed a close correlation between HDL-C and persistent severe SA-AKI (*P* = 0.003) (Fig. 2). The AUC (95% CI) for predicting persistent severe SA-AKI based on HDL-C measured within 24 h of ICU admission was 0.62 (0.56–0.69), with a predicted optimal cutoff value of 31.5 mg/dl, sensitivity of 74%, and specificity of 46% (Fig. 3). Serum creatinine, ΔScr, APSIII, Lactate, Heart rate, and APTT were found to be independently associated with persistent severe SA-AKI based on the results of a multivariate analysis (Fig. 4). A predictive model was constructed using these multiple factors, which demonstrated good performance in predicting persistent severe SA-AKI with an AUROC [95% CI] of 0.88[0.84–0.91]. However, when combined with these parameters, HDL-C was neither independently associated with persistent severe SA-AKI nor did it improve the performance of the clinical model for predicting this condition (Table 3, Additional file 1: Table S1).

**Table 3:** Logistic regression models for the early prediction of persistent severe SA-AKI and KRT

Variable included	Clinical model		Model 2	
	Odds ratio [95% CI]	*p* value	Odds ratio [95% CI]	*p* value
*Persistent severe SA-AKI*				
APSIII	1.03 (1.00–1.04)	0.002	1.06 (1.03–1.09)	< 0.001
Scr (mg/dL)	1.23 (1.03–1.50)	0.027	1.29 (1.13–1.49)	0.024
Lactate (mmol/L)	1.24 (1.05–1.38)	0.003	1.22 (1.07–1.39)	0.003
Heart rate (beats/min)	1.01 (1.00–1.03)	0.05	1.02 (1.00–1.03)	0.047
APTT (s)	1.00 (1.00–1.02)	0.02	1.00 (1.00–1.02)	0.021
ΔScr (mg/dL)	1.90 (1.27–2.97)	0.003	1.92 (1.28–3.20)	0.002
HDL-C (mg/dL)	–	–	1.00 (0.98–1.02)	0.45
AUROC	0.88	AUROC	0.88	
*KRT*				
APSIII	1.02 (1.00–1.06)	0.0378	1.02 (1.00–1.06)	0.0376
Scr (mg/dL)	3.86 (2.47–6.60)	< 0.001	3.87 (2.47–6.66)	< 0.001
Lactate (mmol/L)	1.24 (1.01–1.53)	0.033	1.25 (1.01–1.54)	0.033
Platelets (k/uL)	0.99 (0.98–0.99)	0.009	0.99 (0.97–0.99)	0.01
HDL-C (mg/dL)	–	–	0.99 (0.96–1.03)	0.78
AUROC	0.94	AUROC	0.94	

Data are presented as odds ratios [95% confidence intervals]. *SA-AKI* sepsis associated acute kidney injury; *APS III* Acute Physiology Score III, *ΔScr* changes in serum creatinine within 24 after ICU admission, *HDL-C* high density lipoprotein cholesterol, *Scr* serum creatinine, *KRT* kidney replacement therapy

### Ability of HDL-C to predict KRT

In the included SA-AKI population, there was a significant difference in HDL-C levels between the KRT group and the non-KRT group (51.0 [42.8–59.0] vs. 37.0 [30.0–45.0], *p* < 0.001) (Addtional file 1: Fig. S2D). The HDL-C level measured within 24 h of ICU admission predicted KRT with an AUC (95% CI) of 0.66 (0.58–0.73), with a best cutoff value of 35.5 mg/dl, sensitivity of 65.6%, and specificity of 61.9% (Fig. 3). Multivariate analysis indicated that APS III, Serum creatinine, Lactate and Platelets were independently associated with KRT, and based on these factors, a predictive model was constructed which demonstrated good performance for predicting KRT with an AUROC [95% CI] of 0.94 [0.90–0.97]. Adding HDL-C to this model did not show independent association with KRT nor improve the performance of the clinical model for predicting KRT (Table 3, Additional file 1: Table S1).

## Discussion

The plasma HDL-C levels of patients with persistent severe SA-AKI within 24 h after admission to the ICU were significantly lower than those of patients with non-persistent severe AKI. However, HDL-C showed poor discrimination between persistent severe SA-AKI and non-persistent severe SA-AKI, and did not improve the predictive performance of the clinical model. Our study results do not support the use of plasma HDL-C levels within 24 h after admission to the ICU for identifying persistent severe SA-AKI.

In clinical practice, early identification of persistent severe SA-AKI is of great clinical significance. Early recognition of individuals at risk for persistent AKI and proactive intervention and management can potentially impact the progression of AKI to CKD [5]. Additionally, predicting the short-term reversibility of AKI may help assess the likelihood of needing KRT and ultimately determine the optimal timing to initiate KRT [7]. Previous studies have focused on early identification of persistent renal injury using biomarkers, renal ultrasound, and clinical prediction models [8, 9, 28–30]. Although conflicting results exist in these studies, some progress has been made in early identification of persistent SA-AKI through biomarker-based approaches [29]. Therefore, further exploration into identifying subtypes of SA-AKI based on biomarkers remains an area worth investigating.

High-density lipoprotein (HDL) can stimulate the activity of eNOS through SR-B1, and eNOS is involved in regulating the pathological process that affects the progression of SA-AKI. During sepsis, decreased eNOS activity can lead to microcirculatory dysfunction, which may result in local renal ischemia and contribute to kidney damage and the development of SA-AKI [20]. In a small study involving kidney transplant patients, it was found that among 7 patients with persistent AKI, 6 had reduced eNOS activity in peritubular capillaries isolated from renal biopsy samples. However, among 16 patients with rapid recovery from AKI, only 6 had reduced eNOS activity [31]. This study suggests a correlation between eNOS and the duration of AKI. Another previous study [22] found that compared to patients with normal or high concentrations, those with low HDL-C levels during early sepsis had a 2.8-fold increased risk of developing SA-AKI. Furthermore, HDL-C concentration predicted stages 2–3 SA-AKI with an AUC of 0.754. Although this study did not evaluate the diagnostic performance of HDL-C for diagnosing persistent severe SA-AKI, it did find an independent association between low HDL-C concentration during early sepsis and long-term decline in glomerular filtration rate (adjusted for risk factors including hypertension and diabetes). Based on these findings, we hypothesized that blood HDL-C levels could serve as biomarkers for predicting persistent SA-AKI. However, our research results do not support this hypothesis. We found that plasma HDL-C levels measured within 24 h after ICU admission had an AUC (95% CI) of 0.621 (0.56–0.69) for predicting persistent severe SA-AKI, with a best cutoff value of 31.5 mg/dl, sensitivity of 74%, specificity of 46%. HDL-C was not independently associated with persistent severe SA-AKI and did not improve the predictive performance of the clinical model. Considering the close association between persistent severe SA-AKI and kidney replacement therapy (KRT), we further evaluated the predictive value of blood HDL-C for KRT treatment. Similarly, low HDL-C was not independently associated with KRT and had relatively low clinical efficacy in predicting KRT outcomes.

The HDL-C levels are associated with poor prognosis in sepsis patients. Research has found that during the early stage of sepsis, HDL-C concentration rapidly decreases and whether it recovers or continues to decline affects the survival status of sepsis [32]. In a small-scale study, when the HDL concentration at hospital admission was less than 20 mg/dL, the sensitivity and specificity for predicting 30-day mortality rate were 80% [33]. Transient and persistent SA-AKI may have similar pathophysiological mechanisms [34], and some believe that reversibility of AKI is more related to the severity of kidney damage rather than its mechanism [35]. Our study also found that the SOFA score and APSIIIscore were significantly higher in the persistent SA-AKI group compared to non-persistent severe SA-AKI group, while HDL-C concentration showed a significant negative correlation with severity scores. However, even so, HDL-C still has low predictive efficacy for diagnosing persistent severe SA-AKI.

However, it should be noted that our study only focused on the static value of HDL-C within 24 h after admission to the ICU. This result suggests that HDL-C within 24 h of ICU admission is not very effective in predicting persistent severe SA-AKI. However, our study and previous clinical research results suggest a negative correlation between HDL-C and serum creatinine levels as well as severity scores, and low HDL-C is associated with long-term decline in glomerular filtration rate [22]. These results still indicate that HDL-C may have potential value in predicting persistent AKI, similar to changes in creatinine values. Paying attention to the trend of HDL-C over time may improve the diagnostic value for predicting persistent SA-AKI. However, this study is a retrospective study based on a database and did not collect dynamic changes in HDL-C data. Therefore, further prospective studies are needed to validate the diagnostic value of HDL-C changes for persistent severe SA-AKI. In addition, we excluded 112 patients who had missing plasma creatinine and urine output measurements at 72 h after diagnosis of SA-AKI. This may imply that these patients' renal function has recovered, avoiding multiple serum creatinine measurements or continuous urine output monitoring, which could lead to selection bias. Of course, our study has some highlights. Firstly, our research data are based on a large-scale critical care database with a certain time span and considerable sample size. Furthermore, we specifically focus on the diagnostic value of HDL-C in persistent severe SA-AKI which is an aspect with important clinical significance but less studied by researchers. This expands the knowledge boundaries of HDL-C and provides important references for basic research as well as clinical practice.

## Conclusions

In summary, we have found that the HDL-C concentration within 24 h of ICU admission is not a good indicator for distinguishing between persistent and non-persistent severe SA-AKI, and it does not improve clinical prediction.

### Supplementary Information

**Additional file 1: Table S1.** Integrated discrimination improvement (IDI), category-free net reclassifcation improvement (cfNRI) with the addition of HDL-C. **Fig. S1.** Differences between the high HDL-C group and low HDL-C group. **Fig. S2.** Correlation between HDL-C group and serum creatinine, blood urea nitrogen and differences in HDL-C among different groups.

## Data Availability

Data are available upon reasonable request.
