# Supplementary material for: Low HDL-C can be a biomarker to predict persistent severe AKI in septic patients? A retrospective cohort study
Source: Eur J Med Res. 2023 Dec 5;28:567. doi: 10.1186/s40001-023-01513-9 (PMC10696658; doi:10.1186/s40001-023-01513-9)
Supplement: Supplementary file 1 — Additional file 1: Table S1. Integrated discrimination improvement (IDI), category-free net reclassifcation improvement (cfNRI) with the addition of HDL-C. Fig. S1. Differences between the high HDL-C group and low HDL-C group. Fig. S2. Correlation between HDL-C group and serum creatinine, blood urea nitrogen and differences in HDL-C among different groups. [file 40001_2023_1513_MOESM1_ESM.docx]

**Table S 1** Integrated discrimination improvement (IDI), category-free net reclassifcation improvement (cfNRI) with the addition of HDL-C

|  | Value | [95% CI] | p value |
| --- | --- | --- | --- |
| **Persistent severe SA-AKI** |  |  |  |
| IDI | 0 | 0 | NaN |
| IDI: event | 0 | 0 | NaN |
| IDI: non-event | 0 | 0 | NaN |
| cfNRI | 0.003 | -0.037-0.059 | 0.90 |
| cfNRI:event | 0 | -0.035-0.056 | 0.89 |
| cfNRI:non-event | 0.003 | -0.011-0.013 | 0.61 |
| **KRT** |  |  |  |
| IDI | 0 | 0 | NaN |
| IDI: event | 0 | 0 | NaN |
| IDI: non-event | 0 | 0 | NaN |
| cfNRI | 0.017 | -0.046-0.062 | 0.496 |
| cfNRI:event | 0.015 | -0.046-0.066 | 0.548 |
| cfNRI:non-event | 0.001 | -0.011-0.009 | 0.802 |

SA-AKI,Sepsis associated acute kidney injury;KRT,Kidney replacement therapy;IDI,Integrated discrimination improvement,cfNCI,category-free net reclassifcation improvement;HDL-C,High density lipoprotein cholesterol;


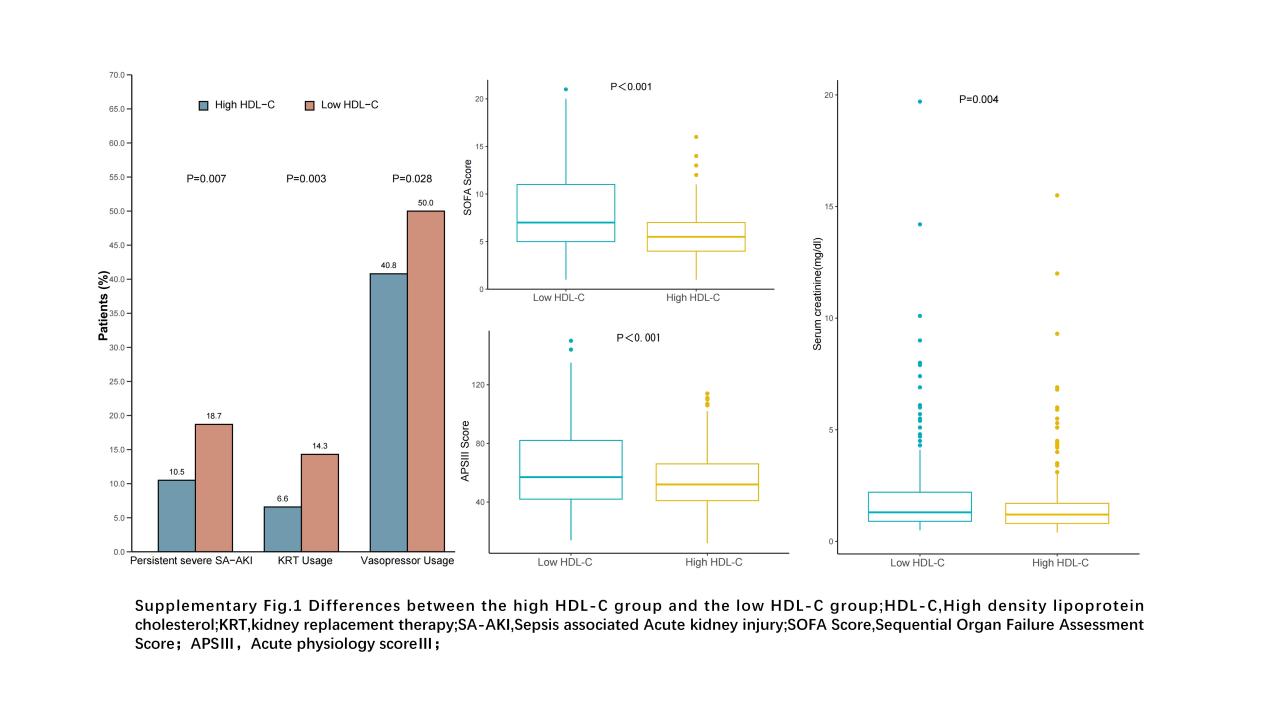


**Fig.S1** Differences between the high HDL-C group and the low HDL-C group;HDL-C,High density lipoprotein cholesterol;KRT,kidney replacement therapy;SA-AKI,Sepsis associated Acute kidney injury;SOFA Score,Sequential Organ Failure AssessmentScore;APSIIl,Acute physiology score lll;


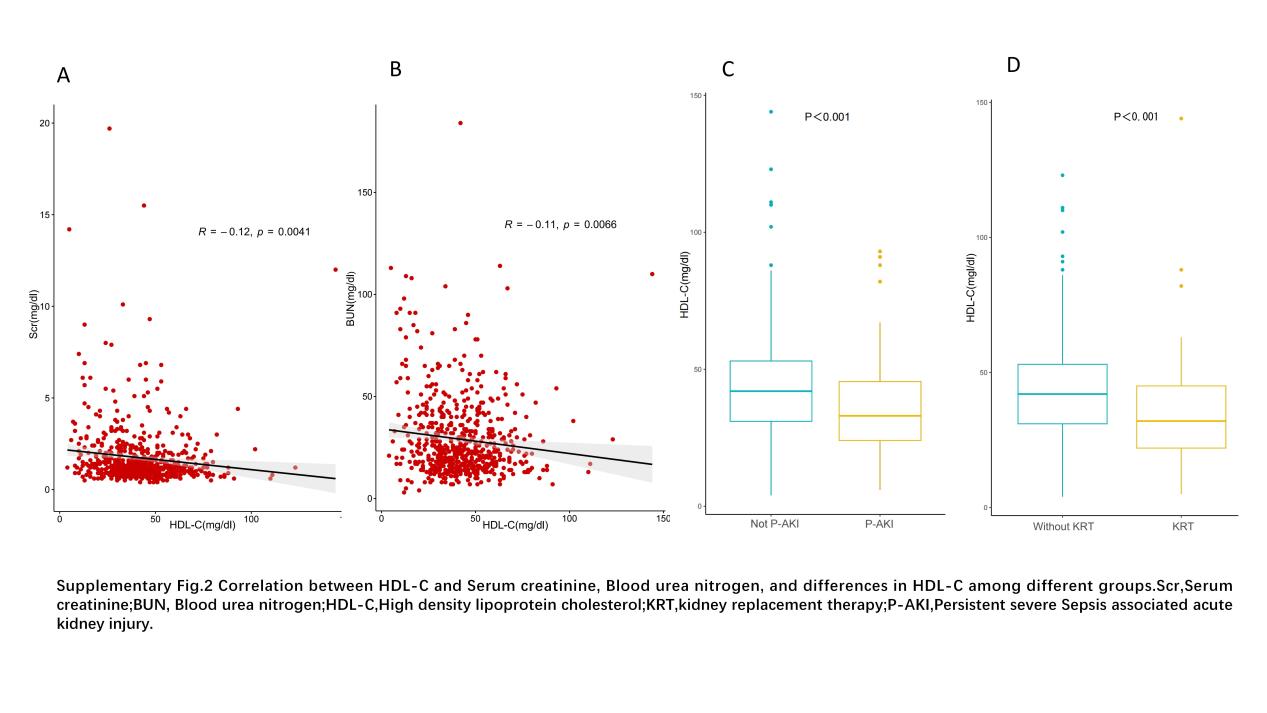


**Fig.S2** Correlation between HDL-C and Serum creatinine,Blood urea nitrogen,and differences in HDL-C among different groups.Scr,Serumcreatinine;BUN,Blood urea nitrogen;HDL-C,High density lipoprotein cholesterol;KRT,kidney replacement therapy;P-AKl,Persistent severe Sepsis associated acutekidney injury.
